# Correlation between the operation time using two different power settings of a Ho: YAG laser: laser power doesn’t influence lithotripsy time

**DOI:** 10.1186/1756-0500-6-80

**Published:** 2013-03-05

**Authors:** Takashi Kawahara, Hiroki Ito, Hideyuki Terao, Yoshitake Kato, Katsuyuki Tanaka, Takehiko Ogawa, Hiroji Uemura, Yoshinobu Kubota, Junichi Matsuzaki

**Affiliations:** 1 Department of Urology, Ohguchi Higashi General Hospital, 2-19-1, Irie, Kanagawa-ku, Yokohama, Kanagawa, JAPAN; 2 Department of Urology, Yokohama City University Graduate School of Medicine, 3-9, Fukuura, Kanazawa-ku, Yokohama, Kanagawa, Japan; 3 Department of Urology, Kanagawa Rehabilitation Hospital, 516, Nanasawa, Atsugi, Kanagawa, Japan

**Keywords:** Cystolithotripsy, Ho, YAG laser, Bladder stone, Bladder calculi

## Abstract

**Background:**

This study investigated the correlation between the operation time using two different power settings of a Ho: YAG laser.

**Findings:**

A total of 68 patients underwent cystolithotripsy from April 2010 to October 2011 In Fifty-six of these patients underwent cystolithotripsy by one surgeon using a Ho: YAG laser for bladder calculi. This study assessed these patients in two groups; the 30 W laser generator group with the settings of 2.5 J x 5 Hz (30 W group) and the 100 W laser generator group as the settings of 3.5 J x 5 Hz (100 W group). The operation time in these two groups were assessed.

A total of 56 patients including 45 male and 11 female patients that underwent cystolithotripsy using a Ho: YAG laser for bladder calculi by one surgeon were enrolled in this study. The patients’ characteristics including age (mean; 68.8 vs 68.4 yr), gender (male; 74.2 vs 88.0%), stone burden (mean; 34.9 vs 41.3 mm), number of stones (mean; 3.2 vs 2.0) and stone’s CT density (mean; 981.5 vs 902.0 HU) showed no significant differences. All patients were stone free following treatment. The median total length of the operation was 19 minutes (mean: 34.6 ± 36.1) in the 30 W group and 29 minutes (mean: 44.4 ± 38.8) in the 100 W group, which was not significantly different.

**Conclusions:**

The results showed that the power settings of Ho: YAG laser show no differences in the operation time for bladder calculi lithotripsy.

## Findings

### Background

Bladder calculi account for 5% of urinary calculi and usually occur because of bladder outlet obstruction (BOO), neurogenic bladder, infection, and foreign bodies [1]. There are several established treatment modalities for bladder stone including shockwave lithotripsy (SWL), transurethral lithotripsy, percutaneous lithotripsy and open surgery [2].

Bladder calculi in adults patients are usually treated endoscopically using litholopaxy, ultrasound lithotripsy, electrohydraulic lithotripsy, pneumatic lithotripsy and a Holmium: Yttrium Aluminum Garnet (Ho: YAG) laser [3,4]. Wide spreading use of Ho: YAG laser for litholisis has confirmed the effectiveness and safety for bladder calculi litholisis [3-7].

In previous reports, the laser settings of ureteroscopic lithotripsy were set at an energy level of 0.5-2.0 J and a rate of 5Hz [8]. Due to recent improvements of Ho: YAG laser generators, there are now available of a wide range of laser settings. However, no detailed laser settings of Ho: YAG laser lithotripsy have yet been established for cystolithotripsy and ureteroscopic lithotripsy. To investigate differences between the power and operation time, we established two different laser settings at the same frequency of 5Hz and investigated the impact of the power of the Ho: YAG laser on the length of the operative time for bladder calculi.

### Methods

A total of 68 patients were underwent cystolithotripsy from April 2010 to October 2011 at our institute. In these patients, 56 patients were included in this study. The exclusion criteria were as follows: 1) Patients who did not agree to give their informed consent, 2) Patients who underwent both cystoscopic lithotripsy and SWL on the same day. All cystolithotripsy procedures were performed by a single surgeon (TK). All bladder calculi fragments that were not made by a Ho: YAG laser lithotripsy

This study was an interventional prospective study. The patients were divided into two groups according to when they underwent cystolithotripsy. Cystolithotripsy using the 30 W Ho: YAG laser generator was performed between April 2010 and March 2011 and cystolithotripsy using the 100 W Ho: YAG laser generator was performed between March 2011 and September 2011. Written informed consent was obtained from all patients and Institutional Review Board of Ohguchi Higashi General Hospital was approved in this study. All patients included in this study were evaluated on both kidney-ureter-bladder (KUB) films and non-contrast computed tomography (NCCT) preoperatively.

All procedures were performed under spinal or general anesthesia in the lithotomy position. A 26 Fr respect scope (Karl Storz, Tuttlingen, Germany) was inserted transurethrally. The Ho: YAG laser were used in making stone fragments. The laser generators used included 30 W or 100 W Ho: YAG lasers (VersaPulse 30 W® and VersaPulse PowerSuite 100 W®, LUMENIS surgical, CA, USA). Cystolithotripsy was performed using a Ho: YAG laser through 550 micrometer laser fibers (SlimLine®, LUMENIS surgical, CA, USA). The settings of the laser generator were 2.5 J with 5Hz in the 30 W group and 3.5 J with 5Hz in the 100 W group. To investigate the power of the laser settings, we set the standard settings for ureteroscopic lithotripsy were set at the same frequency of 5 Hz in our institute [8]. All stone fragments were removed by irrigation through the resection scope lumen.

KUB films and NCCT were performed on the same day as the operation and KUB films were obtained on the postoperative day1. Cystolithotripsy using the 30 W Ho: YAG laser generator was performed between April 2010 and March 2011 and cystolithotripsy using the 100 W Ho: YAG laser generator was performed between March 2011 and September 2011. A chemical analysis of stone fragments was performed.

The operation time was determined in two periods. The total operation time is from inserting cystoscope to concluding urethral catheterization. The operation time during litholisis is from starting litholisis to concluding urethral catheterization. Stone free (0 mm) was determined by postoperative day1 KUB films. Perioperative complications were assessed and scored according to the modified Clavien classification system [9-11]. Elevated fever was also defined as > 38.5°C for more than 3 days.

### Statistical analysis

All continuous variables are expressed as the means ± SD. The numerical data were compared by Student’s *t*-test. Thee correlation between variables was determined by the Spearman correlation analysis. A *P*-value of 0.05 or less was considered to be significant.

### Results

A total of 56 patients including 45 male and 11 female patients treated by one surgeon (TK) that performed cystolithotripsy using a Ho: YAG laser for bladder calculi were enrolled in this study. The patients’ characteristics including age, gender, number of stones, stone size and stone’s CT density in each group are shown in Table 1.

**Table 1:** Patient characteristics

**Variables**	**Number (%) or Median (mean ±SD)**		***P***
	**30 W**	**100 W**	**value**
No. of Pts.	31	25	
Median age (yr)	70 (68.8 ± 16.5)	69 (68.4 ± 12.9)	n.s.
Gender			
Male (%)	23 (74.2%)	22 (88.0%)	n.s.
Female (%)	8 (25.8%)	3 (12.0%)	
Stone burden (mm)	25 (34.9 ± 29.6)	32 (41.3 ± 31.3)	n.s.
Maximum stone’s diameter (mm)	15 (17.9 ± 9.3)	20 (22.4 ± 10.1)	0.09
No. of stones	2 (3.2 ± 3.5)	1 (2.0 ±1.7)	n.s.
Solitary	15 (48.4%)	14 (56.0%)	n.s.
2	7 (22.6%)	6 (24.0%)	
3 or more	9 (29.0%)	5 (20.0%)	
Mean CT density of maximum stone’s center area (HU)	981.5 (995.7 ± 394.4)	902 (915.6 ± 297.3)	n.s.

n.s.: not significant.

Table 2 shows the intraoperative and postoperative outcomes. All patients were stone free following treatment. The median total operation time was 19 minutes (mean: 34.6 ± 36.1) in the 30 W group and 29 minutes (mean: 44.4 ± 38.8) in the 100 W group. The median operation time during litholisis was 17 minutes (mean: 32.6 ± 36.5) in the 30 W group and 30 minutes (mean: 40.7 ± 39.2) in the 100 W group. There was no difference in the total operation time in each group. Four patients had urethral stricture, and cystolithotripsy was performed after incision or dilation of the urethral stricture. This study included three patients (one case in the 30 W group and two cases in the 100 W group) whose stone burden was > 40 mm. Therefore, there were differences between the median and mean value due to these patients with a very high stone burden.

**Table 2:** Intraoperative and postoperative outcomes

**Variables**	**Number (%) or Median (mean ±SD)**		***P***
	**30 W**	**100 W**	**value**
Stone free rate (0 mm)	31 (100.0%)	25 (100.0%)	
Total operation time (min.)	19 (34.6 ± 36.1)	29 (44.4 ± 38.8)	n.s.
Operation time during lithotripsy (min.)	17 (32.6 ± 36.5)	30 (40.7 ± 39.2)	n.s.
Total energy using Ho: YAG laser (kJ)	1.25 (5.99 ± 12.04)	7.28 (18.51 ± 31.26)	0.08
Stone analysis			
Calcium Oxalate	10 (32.2%)	9 (36.0%)	n.s.
Struvite	6 (19.4%)	1 (4.0%)	
Uric acid	2 (6.5%)	6 (24.0%)	
Calcium Phosphate	3 (9.7%)	0 (0.0%)	
Mixed	3 (9.7%)	2 (8.0%)	
Unknown	7 (22.6%)	7 (28.05)	
Intraoperative complications	0 (0.0%)	0 (0.0%)	
Postoperative outcomes			
Clavien grading score I	3 (9.7%)	2 (8.0%)	n.s.
Clavien grading score II or more	0 (0.0%)	0 (0.0%)	n.s.

n.s.: not significant.

No intra and postoperative complications of Clavien grading score two or more were observed. Complications of Clavien grading score one included elevated fever in three patients (5.4%) and urinary retention in two patients (3.6%). All patients were cured conservatory.

Chemical analysis revealed calcium oxalate in 19 (33.9%), struvite in 7 (12.5%), uric acid in 8 (14.3%) and calcium phosphate in 3 (5.4%). Five stones (8.9%) were mixed stones and 14 stones (25.0%) were unknown.

### Discussion

There are several established treatment modalities for bladder stone including shockwave lithotripsy (SWL), transurethral lithotripsy, percutaneous lithotripsy and open surgery [2]. Cystolithotripsy is the standard treatment for bladder calculi in adult patients. Transurethral lithotripsy obtains stone fragments using electrohydraulic lithotripsy (EHL), ultrasonic lithotripsy, pneumatic lithotripsy or a Ho: YAG laser [2]. Many procedures are reported for bladder stones; however, most cases obtain stone fragments using a Ho: YAG laser or pneumatic lithotripsy recently [2,12-16]. The usefulness of Ho: YAG laser lithotripsy is accepted widely, even for large bladder calculi [6]. No evaluation of the operation time and the laser power settings for treating bladder stones using Ho: YAG laser lithotripsy has been reported. This study assessed these correlations with Ho: YAG laser lithotripsy.

The Ho: YAG laser lithotripter is a true thermal laser [17]. It has a wavelength of 2100 nm, at the infrared portion of the light spectrum, creating a microscopic vaporization bubble at the fiber’s tip [18]. The vaporization bubble is able to destabilize or “vaporize” tissue or stones [17]. The depth of thermal injury to the tissue in contact on laser activation is 0.5 – 1 mm, which limits the possibility of deep thermal injury to tissue [4].

Previous reports used laser settings of 0.5 to 2.0 J with a frequency 5 to 20 Hz [3,4,6]. The power was increased if the stone fragments are difficult make. The laser power was set from 2.5 J in the 30 W group and 3.5 J in the 100 W group and the frequency was fixed in 5Hz in the current series. The laser power was higher in comparison to the previous studies, but no mucosal injuries were observed. Higher total energy was obtained in 100 W group, however, there was no decrease in the operation time. This suggested that that the power of laser lithotripsy is high and stone fragmentation is easily obtained in low power settings.

The operation time in ureteroscopic lithotripsy is influenced by multiple factors including using a urethral access sheath, preoperative stenting, using a flexible or rigid ureteroscopy and stone location [19,20]. The correlation between the laser power and operation time suggests that cystolithotripsy is better than ureteroscopic lithotripsy and percutaneous lithotripsy.

The stone size, stone hardness and laser power are probably the main factors affecting the cystolithotripsy operation time. Urethral strictures were seen in some male patients. Therefore, the time was assessed from the beginning of lithotripsy to concluding catheterization for the purpose of decreasing the factor of gender. However there was still no difference in the operation time.

This study has limitations associated with the laser settings. We set the intermission as 5Hz because that is the setting used in Ureteroscopic lithotripsy. We did not change the rate during the operation in this study for evaluating the power of laser energy. The rate of intermission was set to 8Hz in some cases that were not included in this study, and that appeared to affect the time. In our institute, we set different rate settings for bladder stone lithotripsy, and in some cases, the higher rate allowed for a decrease in the length of the operation time. However, because of the low visibility, some patients needed higher irrigation flow. Therefore, we uset the lower rate (5 Hz) setting in this study. Based upon the observed safety for the higher power laser settings, an additional study is needed to determine whether it is possible to decrease the length of the operation when using higher power and higher rates for the laser settings.

### Conclusions

The results showed that stone’s area and volume are correlated with the operation time more than the stone burden for bladder calculi litholisis using the Ho: YAG laser.

## Abbreviations

BOO: Bladder outlet obstruction;Ho: Holmium;YAG: Yttrium Aluminum Garnet;SWL: Shockwave lithotripsy;KUB: Kidney-ureter-bladder;NCCT: Non-contrast computed tomography

## Competing interests

The authors declare that they have no competing interests.

## Authors’ contributions

TK, HI, HT, YK and JM analyzed the patients’ data regarding operation procedure. TK, KT, TO, HU and YK wrote the manuscript. All authors read and approved the final manuscript.

## Concise description of the reported work

This study investigated the correlation between the operation time using two different power settings of a Ho: YAG laser lithotripsy. A total of 68 patients underwent cystolithotripsy with the settings of 2.5 J x 5 Hz (30 W group) and the 100 W laser generator group as the settings of 3.5 J x 5 Hz (100 W group). The median total length of the operation was 19 minutes (mean: 34.6 ± 36.1) in the 30 W group and 29 minutes (mean: 44.4 ± 38.8) in the 100 W group, which was not significantly different. The results showed that the power settings of Ho: YAG laser show no differences in the operation time for bladder calculi lithotripsy.
